# Supplementary material for: Evolution favors protein mutational robustness in sufficiently large populations
Source: BMC Biol. 2007 Jul 17;5:29. doi: 10.1186/1741-7007-5-29 (PMC1995189; doi:10.1186/1741-7007-5-29)
Supplement: Additional file 3 — Thermostability measurements. Raw data from the T50 thermostability measurements. [file 1741-7007-5-29-S3.pdf]

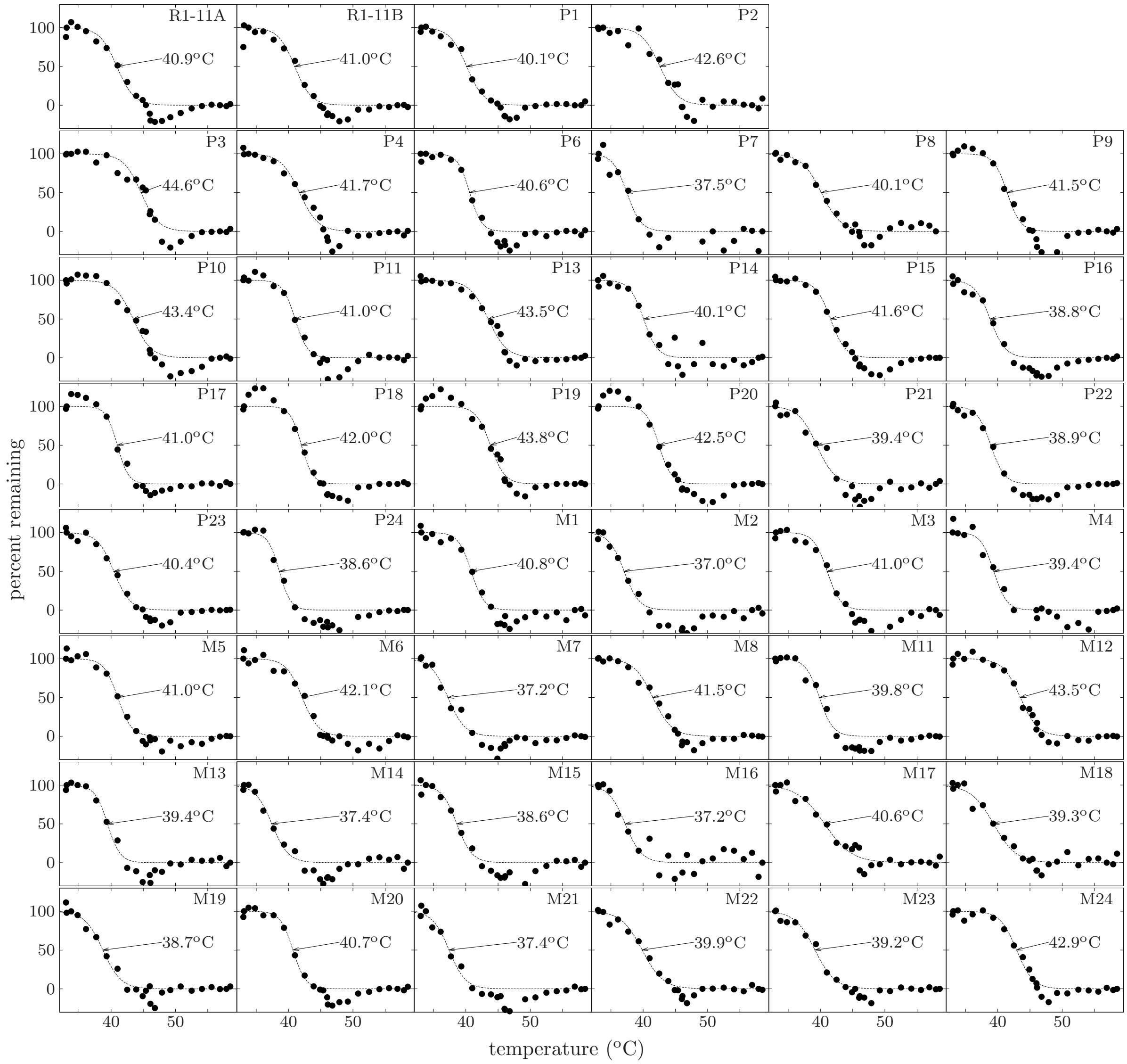

Thermostability measurements. The stability to irreversible thermal denaturation was quantified as T_{50}, the temperature at which half of the protein irreversibly denatured after a 10 minute incubation. Curves show the percent of the protein remaining after incubation at the indicated temperatures. The T_{50} was determined by fitting the data to a sigmoidal curve of the form $f(t) = 100/\left(1 + e^{a(T - T_{50})}\right)$ where T is the temperature and $f(T)$ is the percent of protein remaining at temperature T.
