# Supplementary material for: Evolution favors protein mutational robustness in sufficiently large populations
Source: BMC Biol. 2007 Jul 17;5:29. doi: 10.1186/1741-7007-5-29 (PMC1995189; doi:10.1186/1741-7007-5-29)
Supplement: Additional file 4 — Urea stability measurements. Raw data from the [urea]50 thermostability measurements. [file 1741-7007-5-29-S4.pdf]

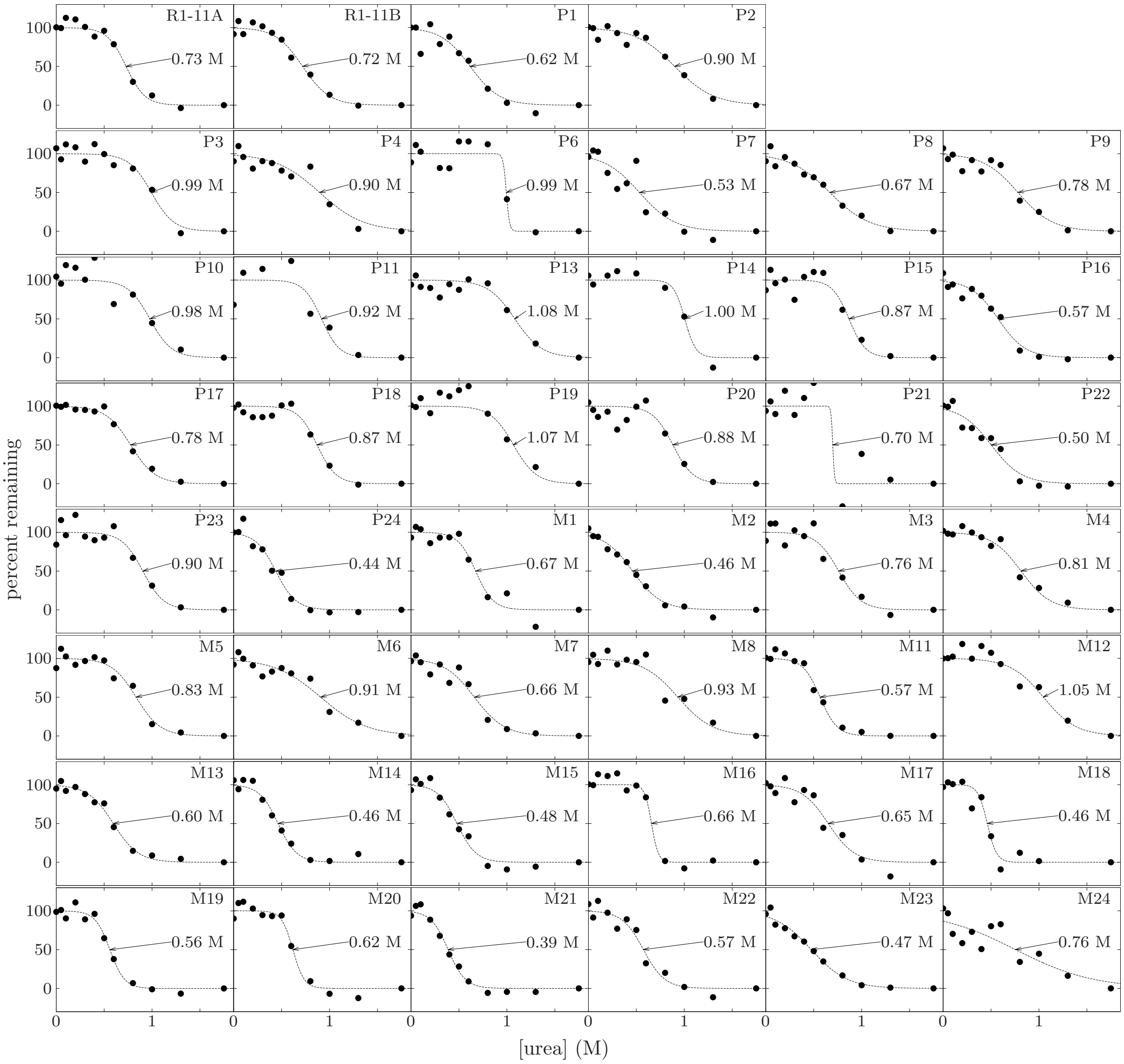

Urea stability measurements. The stability to irreversible urea denaturation was quantified as the $[\text{urea}]_{50}$, the urea concentration at which half of the protein irreversibly denatured after a 4 hour incubation at room temperature. Curves show the percent of the protein remaining after incubation at the indicated urea concentrations. The $[\text{urea}]_{50}$ was determined by fitting the data to a sigmoidal curve of the form $f(u) = 100/\left(1 + e^{a\left(u - [\text{urea}]_{50}\right)}\right)$ where u is the urea concentration and $f(u)$ is the percent of protein remaining at urea concentration u.
