# Supplementary material for: Evolution favors protein mutational robustness in sufficiently large populations
Source: BMC Biol. 2007 Jul 17;5:29. doi: 10.1186/1741-7007-5-29 (PMC1995189; doi:10.1186/1741-7007-5-29)
Supplement: Additional file 5 — Correlation of thermal and urea stabilities. The T50 and [urea]50 values are highly correlated. [file 1741-7007-5-29-S5.pdf]

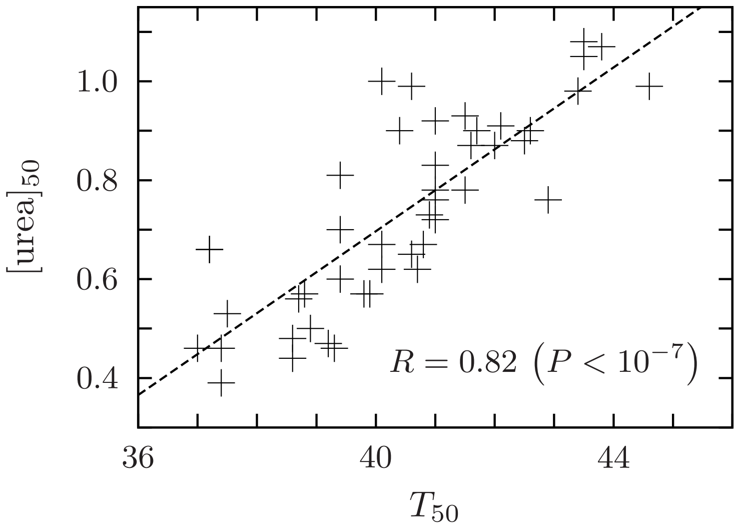

Correlation of thermal and urea stabilities. The T_{50} and $[\text{urea}]_{50}$ stability measurements are highly correlated, indicating that they consistently measure a universal aspect of protein stability. The Pearson correlation coefficient is $R = 0.82$, with a probability of $P \approx 10^{-7}$ that a correlation this large would be observed among the 46 measurements by chance.
